# Tetra-μ_2_-acetato-diaqua­bis­(μ_2_-2-{[1,3-dihy­droxy-2-(oxidometh­yl)propan-2-yl]imino­meth­yl}phenolato)trimanganese(II,III) acetonitrile disolvate dihydrate

**DOI:** 10.1107/S1600536811027899

**Published:** 2011-07-16

**Authors:** Yuhua Guo, Jianping Huang, Yong Huang, Junyue Wang, Youzhu Yu

**Affiliations:** a Department of Chemistry and Environmental Engineering, Anyang Institute of Technology, Henan 455000, People’s Republic of China

## Abstract

In the title complex, [Mn^II^Mn^III^_2_(C_11_H_13_NO_4_)_2_(CH_3_CO_2_)_4_(H_2_O)_2_]·2CH_3_CN·2H_2_O, there are two Mn^III^ and one Mn^II^ atoms. The Mn^II^ atom lies on an inversion center and the Mn^III^—Mn^II^—Mn^III^ angle is therefore 180°, as required by crystallographic symmetry. The Mn^III^ and Mn^II^ atoms are six-coordinated in a distorted octa­hedral geometry. In the crystal, complex mol­ecules and solvent mol­ecules are linked into a three-dimensional network by O—H⋯O and O—H⋯N hydrogen-bonding inter­actions.

## Related literature

For the importance of Mn complexes in magnetism and biomimetics, see: Stamatatos & Christou (2009 ▶); Ferreira *et al.* (2004 ▶). For properties and structures of related compounds, see: Kessissoglou *et al.* (1992 ▶); Liu *et al.* (2010 ▶). 
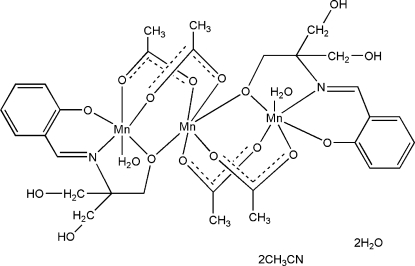


## Experimental

### 

#### Crystal data

[Mn_3_(C_11_H_13_NO_4_)_2_(C_2_H_3_O_2_)_4_(H_2_O)_2_]·2C_2_H_3_N·2H_2_O
*M* *_r_* = 1001.62
Monoclinic, 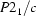

*a* = 10.6032 (5) Å
*b* = 12.2114 (6) Å
*c* = 19.1608 (9) Å
β = 118.856 (3)°
*V* = 2172.89 (18) Å^3^
*Z* = 2
Mo *K*α radiation
μ = 0.94 mm^−1^
*T* = 293 K
0.20 × 0.20 × 0.20 mm

#### Data collection

Bruker APEXII CCD diffractometer
53356 measured reflections
5507 independent reflections
3798 reflections with *I* > 2σ(*I*)
*R*_int_ = 0.086

#### Refinement

*R*[*F* ^2^ > 2σ(*F* ^2^)] = 0.047
*wR*(*F* ^2^) = 0.133
*S* = 1.07
5507 reflections
294 parameters
7 restraints
H atoms treated by a mixture of independent and constrained refinement
Δρ_max_ = 0.72 e Å^−3^
Δρ_min_ = −0.61 e Å^−3^

### 

Data collection: *APEX2* (Bruker, 1996 ▶); cell refinement: *SAINT* (Bruker, 1996 ▶); data reduction: *SAINT*; program(s) used to solve structure: *SHELXS97* (Sheldrick, 2008 ▶); program(s) used to refine structure: *SHELXL97* (Sheldrick, 2008 ▶); molecular graphics: *SHELXTL* (Sheldrick, 2008 ▶); software used to prepare material for publication: *SHELXTL*.

## Supplementary Material

Crystal structure: contains datablock(s) I, global. DOI: 10.1107/S1600536811027899/pv2425sup1.cif

Structure factors: contains datablock(s) I. DOI: 10.1107/S1600536811027899/pv2425Isup2.hkl

Additional supplementary materials:  crystallographic information; 3D view; checkCIF report

## Figures and Tables

**Table 1:** Hydrogen-bond geometry (Å, °)

*D*—H⋯*A*	*D*—H	H⋯*A*	*D*⋯*A*	*D*—H⋯*A*
O9—H9*A*⋯O10^i^	0.85 (1)	1.97 (1)	2.806 (4)	167 (3)
O9—H9*B*⋯O8^ii^	0.85 (1)	2.23 (3)	3.008 (3)	153 (5)
O9—H9*B*⋯O1^ii^	0.85 (1)	2.61 (3)	3.322 (3)	142 (5)
O10—H10*C*⋯O5^iii^	0.85 (1)	2.06 (1)	2.907 (4)	176 (5)
O10—H10*D*⋯N2^iv^	0.85 (1)	2.07 (1)	2.914 (6)	174 (6)
O2—H2⋯O3^v^	0.82	2.55	3.362 (5)	172
O3—H3⋯O6^vi^	0.82	2.00	2.777 (3)	159

Symmetry codes: (i) 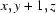; (ii) 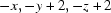; (iii) 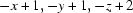; (iv) 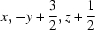; (v) 
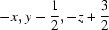
; (vi) 
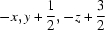
.

**Fig. 1.**
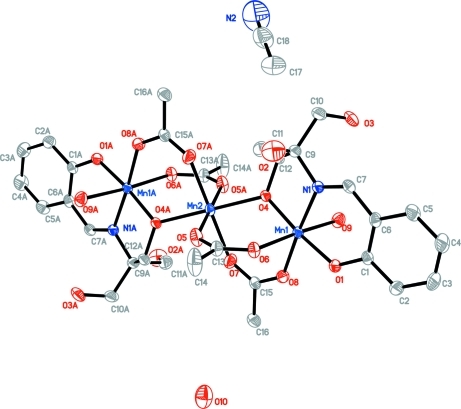
The molecular structure of the title compound, with atom labels and 30% probability displacement ellipsoids.

## supplementary crystallographic information

### Comment

The fascination of inorganic chemists with Mn coordination chemistry over the last two decades or so has been primarily driven by the relevance of Mn-complexes to magnetic and biomimetic fields (Stamatatos & Christou, 2009; Ferreira *et al.*2004). As a contribution to these fields, we report here the synthesis and crystal structure of the title compound.

In the title complex (Fig. 1), the Mn^III^ and Mn^II^ atoms are six-coordinated in a distorted octahedral geometry and the two Mn^III^ are in the same coordination environment. The Mn(II) lies on an inversion center, therefore, the angle  Mn(III)-Mn(II)—Mn(III) is 180° as required by crystallographic symmetry. The bond lengths and bond angles in the title complex are comparable with those observed in the related complexes (Kessissoglou *et al.*, 1992). In the crystal structure, the complex molecules and the solvent molecules are linked through intermolecular O—H···O and O—H···N hydrogen bonds (Table 1) into a three-dimensional network.

### Experimental

To a stirred acetonitrile (20 ml) solution of H_2_SALATHM (1 mmol, 225 mg) was added Mn(OAc)_2_.4H_2_O (1 mmol, 245 mg). The resulting dark-red solution was stirred for 1 h and the filtrate was allowed to stand at room temperature for about three days, whereupon dark block crystal suitable for X-ray diffraction analysis was obtained.

### Refinement

H atoms were placed at calculated positions with O–H = 0.82 Å (hydroxyl), and C—H = 0.93 Å (aryl), 0.97 (methylene) and 0.96 Å (methyl) and were refined in the riding-model approximation with *U*_iso_ = 1.2–1.5 times *U*_eq_ of the parent atoms. The H-atoms of water of solvation were located from a difference map and were included at distances 0.85 (1) using DFIX commands and were allowed *U*_iso_ = 1.5 times *U*_eq_(O).

### Crystal data

**Table:**

[Mn_3_(C_11_H_13_NO_4_)_2_(C_2_H_3_O_2_)_4_(H_2_O)_2_]·2C_2_H_3_N·2H_2_O	*F*(000) = 1038
*M**_r_* = 1001.62	*D*_x_ = 1.531 Mg m^−^^3^
Monoclinic, *P*2_1_/*c*	Mo *K*α radiation, λ = 0.71073 Å
Hall symbol: -P 2ybc	Cell parameters from 9948 reflections
*a* = 10.6032 (5) Å	θ = 2.4–28.4°
*b* = 12.2114 (6) Å	µ = 0.94 mm^−^^1^
*c* = 19.1608 (9) Å	*T* = 293 K
β = 118.856 (3)°	Block, black
*V* = 2172.89 (18) Å^3^	0.20 × 0.20 × 0.20 mm
*Z* = 2	

### Data collection

**Table:**

Bruker APEXII CCD diffractometer	3798 reflections with *I* > 2σ(*I*)
Radiation source: fine-focus sealed tube	*R*_int_ = 0.086
graphite	θ_max_ = 28.6°, θ_min_ = 2.1°
φ and ω scans	*h* = −14→14
53356 measured reflections	*k* = −16→16
5507 independent reflections	*l* = −25→25

### Refinement

**Table:**

Refinement on *F*^2^	Primary atom site location: structure-invariant direct methods
Least-squares matrix: full	Secondary atom site location: difference Fourier map
*R*[*F*^2^ > 2σ(*F*^2^)] = 0.047	Hydrogen site location: inferred from neighbouring sites
*wR*(*F*^2^) = 0.133	H atoms treated by a mixture of independent and constrained refinement
*S* = 1.07	*w* = 1/[σ^2^(*F*_o_^2^) + (0.0525*P*)^2^ + 2.7933*P*] where *P* = (*F*_o_^2^ + 2*F*_c_^2^)/3
5507 reflections	(Δ/σ)_max_ = 0.024
294 parameters	Δρ_max_ = 0.72 e Å^−^^3^
7 restraints	Δρ_min_ = −0.61 e Å^−^^3^

### Fractional atomic coordinates and isotropic or equivalent isotropic displacement parameters (Å^2^)

**Table:**

	*x*	*y*	*z*	*U*_iso_*/*U*_eq_	
C1	−0.1569 (3)	0.8511 (2)	0.84008 (18)	0.0317 (6)	
C2	−0.2687 (4)	0.8090 (3)	0.8512 (2)	0.0419 (8)	
H2A	−0.2506	0.7888	0.9020	0.050*	
C3	−0.4053 (4)	0.7971 (3)	0.7877 (2)	0.0548 (10)	
H3A	−0.4788	0.7710	0.7966	0.066*	
C4	−0.4352 (4)	0.8235 (4)	0.7105 (3)	0.0604 (11)	
H4	−0.5274	0.8140	0.6680	0.072*	
C5	−0.3275 (4)	0.8635 (3)	0.6980 (2)	0.0475 (9)	
H5	−0.3469	0.8808	0.6465	0.057*	
C6	−0.1874 (3)	0.8789 (2)	0.76201 (18)	0.0345 (7)	
C7	−0.0836 (3)	0.9291 (2)	0.74451 (18)	0.0334 (6)	
H7	−0.1103	0.9400	0.6911	0.040*	
C9	0.1393 (3)	1.0157 (3)	0.77120 (18)	0.0352 (7)	
C10	0.0585 (4)	1.0849 (3)	0.6962 (2)	0.0464 (9)	
H10A	0.1265	1.1269	0.6868	0.056*	
H10B	0.0049	1.0376	0.6506	0.056*	
C11	0.2297 (4)	0.9275 (3)	0.7590 (2)	0.0486 (9)	
H11A	0.2880	0.9621	0.7388	0.058*	
H11B	0.2946	0.8953	0.8102	0.058*	
C12	0.2357 (3)	1.0870 (3)	0.84214 (18)	0.0342 (7)	
H12A	0.3199	1.1091	0.8382	0.041*	
H12B	0.1840	1.1525	0.8422	0.041*	
C13	0.3707 (3)	0.7594 (2)	0.94069 (19)	0.0357 (7)	
C14	0.3997 (5)	0.6436 (3)	0.9255 (4)	0.0821 (17)	
H14A	0.4021	0.6404	0.8761	0.123*	
H14B	0.3248	0.5965	0.9226	0.123*	
H14C	0.4907	0.6200	0.9682	0.123*	
C15	0.3292 (3)	0.9424 (2)	1.09183 (17)	0.0315 (6)	
C16	0.3403 (4)	0.9114 (3)	1.1703 (2)	0.0440 (8)	
H16A	0.4374	0.9225	1.2120	0.066*	
H16B	0.3147	0.8358	1.1690	0.066*	
H16C	0.2761	0.9561	1.1801	0.066*	
C17	0.1100 (6)	0.8401 (4)	0.5308 (3)	0.0706 (13)	
H17B	0.0142	0.8391	0.4864	0.106*	
H17A	0.1057	0.8323	0.5794	0.106*	
H17C	0.1644	0.7806	0.5258	0.106*	
C18	0.1775 (5)	0.9407 (4)	0.5317 (3)	0.0742 (14)	
H9A	0.082 (4)	1.158 (2)	0.938 (3)	0.111*	
H9B	−0.014 (5)	1.099 (3)	0.953 (3)	0.111*	
H10C	0.320 (2)	0.242 (4)	0.984 (3)	0.111*	
H10D	0.235 (5)	0.327 (3)	0.987 (3)	0.111*	
N1	0.0433 (3)	0.9604 (2)	0.79651 (14)	0.0299 (5)	
N2	0.2282 (7)	1.0199 (4)	0.5303 (5)	0.146 (3)	
O1	−0.0288 (2)	0.86304 (18)	0.90298 (12)	0.0356 (5)	
O2	0.1473 (4)	0.8430 (3)	0.7059 (2)	0.0775 (9)	
H2	0.1298	0.7957	0.7305	0.116*	
O3	−0.0374 (3)	1.1568 (2)	0.70565 (16)	0.0569 (7)	
H3	−0.0849	1.1909	0.6642	0.085*	
O4	0.2792 (2)	1.02733 (16)	0.91436 (11)	0.0290 (4)	
O5	0.4759 (2)	0.82092 (18)	0.97622 (15)	0.0466 (6)	
O6	0.2412 (2)	0.78562 (16)	0.91590 (13)	0.0359 (5)	
O7	0.4363 (2)	0.9780 (2)	1.08976 (14)	0.0480 (6)	
O8	0.2062 (2)	0.92758 (18)	1.03081 (12)	0.0358 (5)	
O9	0.0281 (3)	1.10148 (19)	0.92495 (16)	0.0451 (6)	
O10	0.2353 (3)	0.2668 (3)	0.9640 (2)	0.0640 (8)	
Mn1	0.13180 (4)	0.94343 (3)	0.91490 (2)	0.02691 (13)	
Mn2	0.5000	1.0000	1.0000	0.02878 (16)	

### Atomic displacement parameters (Å^2^)

**Table:**

	*U*^11^	*U*^22^	*U*^33^	*U*^12^	*U*^13^	*U*^23^
C1	0.0261 (14)	0.0260 (14)	0.0358 (16)	−0.0014 (11)	0.0091 (12)	−0.0011 (11)
C2	0.0379 (17)	0.0424 (18)	0.0430 (19)	−0.0061 (14)	0.0176 (15)	0.0013 (14)
C3	0.0326 (17)	0.061 (2)	0.064 (3)	−0.0180 (17)	0.0181 (17)	−0.0055 (19)
C4	0.0317 (18)	0.069 (3)	0.057 (2)	−0.0176 (18)	0.0032 (17)	−0.003 (2)
C5	0.0363 (18)	0.050 (2)	0.0377 (19)	−0.0101 (15)	0.0030 (14)	−0.0016 (15)
C6	0.0258 (14)	0.0306 (15)	0.0382 (17)	−0.0024 (12)	0.0085 (12)	−0.0031 (12)
C7	0.0313 (15)	0.0351 (16)	0.0260 (14)	0.0012 (12)	0.0075 (12)	0.0006 (12)
C9	0.0302 (15)	0.0398 (16)	0.0328 (16)	−0.0026 (13)	0.0129 (13)	0.0056 (13)
C10	0.0397 (18)	0.055 (2)	0.0352 (18)	−0.0057 (16)	0.0104 (15)	0.0132 (15)
C11	0.049 (2)	0.059 (2)	0.042 (2)	0.0034 (17)	0.0253 (17)	−0.0003 (16)
C12	0.0273 (14)	0.0342 (15)	0.0340 (16)	−0.0017 (12)	0.0092 (12)	0.0082 (12)
C13	0.0308 (15)	0.0274 (14)	0.0443 (18)	−0.0002 (12)	0.0145 (14)	−0.0049 (13)
C14	0.045 (2)	0.040 (2)	0.139 (5)	0.0000 (17)	0.026 (3)	−0.030 (3)
C15	0.0321 (15)	0.0281 (14)	0.0313 (15)	0.0043 (12)	0.0130 (12)	−0.0022 (12)
C16	0.0445 (19)	0.0471 (19)	0.0348 (18)	0.0055 (15)	0.0148 (15)	0.0028 (14)
C17	0.102 (4)	0.049 (2)	0.069 (3)	−0.006 (2)	0.047 (3)	−0.004 (2)
C18	0.074 (3)	0.047 (2)	0.087 (3)	0.001 (2)	0.027 (3)	0.011 (2)
N1	0.0262 (12)	0.0323 (13)	0.0284 (12)	−0.0012 (10)	0.0110 (10)	0.0024 (10)
N2	0.124 (5)	0.065 (3)	0.225 (8)	−0.021 (3)	0.065 (5)	0.031 (4)
O1	0.0247 (10)	0.0422 (12)	0.0327 (11)	−0.0046 (9)	0.0080 (9)	0.0057 (9)
O2	0.101 (3)	0.072 (2)	0.068 (2)	−0.0063 (19)	0.047 (2)	−0.0187 (17)
O3	0.0442 (14)	0.0612 (17)	0.0493 (15)	0.0126 (12)	0.0099 (12)	0.0258 (13)
O4	0.0218 (9)	0.0311 (10)	0.0286 (10)	0.0003 (8)	0.0078 (8)	0.0061 (8)
O5	0.0310 (11)	0.0290 (11)	0.0633 (16)	−0.0016 (9)	0.0096 (11)	−0.0065 (11)
O6	0.0285 (10)	0.0283 (10)	0.0440 (13)	−0.0011 (8)	0.0120 (9)	−0.0063 (9)
O7	0.0313 (12)	0.0740 (17)	0.0372 (13)	−0.0035 (12)	0.0152 (10)	−0.0003 (12)
O8	0.0306 (11)	0.0434 (12)	0.0291 (11)	−0.0049 (9)	0.0109 (9)	−0.0004 (9)
O9	0.0436 (14)	0.0384 (13)	0.0596 (16)	0.0039 (10)	0.0299 (12)	−0.0016 (11)
O10	0.0495 (16)	0.0581 (18)	0.081 (2)	0.0004 (13)	0.0286 (16)	−0.0148 (15)
Mn1	0.0215 (2)	0.0281 (2)	0.0258 (2)	−0.00170 (16)	0.00718 (17)	0.00117 (17)
Mn2	0.0204 (3)	0.0280 (3)	0.0314 (3)	−0.0005 (2)	0.0073 (2)	−0.0008 (2)

### Geometric parameters (Å, °)

**Table:**

C1—O1	1.319 (3)	C14—H14B	0.9600
C1—C2	1.400 (4)	C14—H14C	0.9600
C1—C6	1.411 (4)	C15—O7	1.234 (4)
C2—C3	1.378 (5)	C15—O8	1.276 (3)
C2—H2A	0.9300	C15—C16	1.498 (4)
C3—C4	1.393 (6)	C16—H16A	0.9600
C3—H3A	0.9300	C16—H16B	0.9600
C4—C5	1.365 (5)	C16—H16C	0.9600
C4—H4	0.9300	C17—C18	1.418 (6)
C5—C6	1.410 (4)	C17—H17B	0.9600
C5—H5	0.9300	C17—H17A	0.9600
C6—C7	1.434 (4)	C17—H17C	0.9600
C7—N1	1.287 (4)	C18—N2	1.113 (6)
C7—H7	0.9300	N1—Mn1	2.004 (2)
C9—N1	1.484 (4)	O1—Mn1	1.882 (2)
C9—C12	1.520 (4)	O2—H2	0.8200
C9—C10	1.525 (4)	O3—H3	0.8200
C9—C11	1.533 (5)	O4—Mn1	1.8729 (19)
C10—O3	1.420 (5)	O4—Mn2	2.1395 (18)
C10—H10A	0.9700	O5—Mn2	2.223 (2)
C10—H10B	0.9700	O6—Mn1	2.244 (2)
C11—O2	1.416 (5)	O7—Mn2	2.147 (2)
C11—H11A	0.9700	O8—Mn1	1.975 (2)
C11—H11B	0.9700	O9—Mn1	2.275 (2)
C12—O4	1.429 (3)	O9—H9A	0.851 (10)
C12—H12A	0.9700	O9—H9B	0.851 (10)
C12—H12B	0.9700	O10—H10C	0.851 (10)
C13—O5	1.241 (4)	O10—H10D	0.849 (10)
C13—O6	1.258 (4)	Mn2—O4^i^	2.1395 (18)
C13—C14	1.505 (5)	Mn2—O7^i^	2.147 (2)
C14—H14A	0.9600	Mn2—O5^i^	2.223 (2)
			
O1—C1—C2	118.4 (3)	H16A—C16—H16B	109.5
O1—C1—C6	123.4 (3)	C15—C16—H16C	109.5
C2—C1—C6	118.2 (3)	H16A—C16—H16C	109.5
C3—C2—C1	120.7 (3)	H16B—C16—H16C	109.5
C3—C2—H2A	119.7	C18—C17—H17B	109.5
C1—C2—H2A	119.7	C18—C17—H17A	109.5
C2—C3—C4	121.1 (3)	H17B—C17—H17A	109.5
C2—C3—H3A	119.4	C18—C17—H17C	109.5
C4—C3—H3A	119.4	H17B—C17—H17C	109.5
C5—C4—C3	119.3 (3)	H17A—C17—H17C	109.5
C5—C4—H4	120.4	N2—C18—C17	178.1 (7)
C3—C4—H4	120.4	C7—N1—C9	120.5 (3)
C4—C5—C6	120.9 (3)	C7—N1—Mn1	126.1 (2)
C4—C5—H5	119.6	C9—N1—Mn1	113.37 (18)
C6—C5—H5	119.6	C1—O1—Mn1	129.90 (19)
C5—C6—C1	119.8 (3)	C11—O2—H2	109.5
C5—C6—C7	117.3 (3)	C10—O3—H3	109.5
C1—C6—C7	122.8 (3)	C12—O4—Mn1	113.83 (16)
N1—C7—C6	125.4 (3)	C12—O4—Mn2	122.99 (17)
N1—C7—H7	117.3	Mn1—O4—Mn2	121.17 (9)
C6—C7—H7	117.3	C13—O5—Mn2	133.8 (2)
N1—C9—C12	103.9 (2)	C13—O6—Mn1	133.38 (19)
N1—C9—C10	113.5 (3)	C15—O7—Mn2	136.1 (2)
C12—C9—C10	110.8 (3)	C15—O8—Mn1	133.9 (2)
N1—C9—C11	108.0 (3)	Mn1—O9—H9A	116 (2)
C12—C9—C11	109.8 (3)	Mn1—O9—H9B	116 (3)
C10—C9—C11	110.7 (3)	H9A—O9—H9B	109 (2)
O3—C10—C9	109.5 (3)	H10C—O10—H10D	110 (3)
O3—C10—H10A	109.8	O4—Mn1—O1	173.45 (9)
C9—C10—H10A	109.8	O4—Mn1—O8	100.27 (9)
O3—C10—H10B	109.8	O1—Mn1—O8	86.11 (9)
C9—C10—H10B	109.8	O4—Mn1—N1	82.72 (9)
H10A—C10—H10B	108.2	O1—Mn1—N1	90.84 (9)
O2—C11—C9	114.0 (3)	O8—Mn1—N1	176.23 (10)
O2—C11—H11A	108.8	O4—Mn1—O6	92.33 (8)
C9—C11—H11A	108.8	O1—Mn1—O6	89.24 (9)
O2—C11—H11B	108.8	O8—Mn1—O6	88.97 (9)
C9—C11—H11B	108.8	N1—Mn1—O6	93.22 (9)
H11A—C11—H11B	107.6	O4—Mn1—O9	88.58 (9)
O4—C12—C9	109.7 (2)	O1—Mn1—O9	90.39 (9)
O4—C12—H12A	109.7	O8—Mn1—O9	86.27 (9)
C9—C12—H12A	109.7	N1—Mn1—O9	91.54 (10)
O4—C12—H12B	109.7	O6—Mn1—O9	175.23 (9)
C9—C12—H12B	109.7	O4^i^—Mn2—O4	180.000 (1)
H12A—C12—H12B	108.2	O4^i^—Mn2—O7^i^	89.03 (8)
O5—C13—O6	125.6 (3)	O4—Mn2—O7^i^	90.98 (8)
O5—C13—C14	117.5 (3)	O4^i^—Mn2—O7	90.97 (8)
O6—C13—C14	117.0 (3)	O4—Mn2—O7	89.02 (8)
C13—C14—H14A	109.5	O7^i^—Mn2—O7	180.000 (1)
C13—C14—H14B	109.5	O4^i^—Mn2—O5	88.78 (8)
H14A—C14—H14B	109.5	O4—Mn2—O5	91.22 (8)
C13—C14—H14C	109.5	O7^i^—Mn2—O5	90.48 (10)
H14A—C14—H14C	109.5	O7—Mn2—O5	89.52 (10)
H14B—C14—H14C	109.5	O4^i^—Mn2—O5^i^	91.22 (8)
O7—C15—O8	124.7 (3)	O4—Mn2—O5^i^	88.78 (8)
O7—C15—C16	119.5 (3)	O7^i^—Mn2—O5^i^	89.52 (10)
O8—C15—C16	115.7 (3)	O7—Mn2—O5^i^	90.48 (10)
C15—C16—H16A	109.5	O5—Mn2—O5^i^	180.000 (1)
C15—C16—H16B	109.5		
			
O1—C1—C2—C3	178.6 (3)	Mn2—O4—Mn1—O6	−51.58 (12)
C6—C1—C2—C3	−1.2 (5)	C12—O4—Mn1—O9	−71.9 (2)
C1—C2—C3—C4	1.9 (6)	Mn2—O4—Mn1—O9	123.73 (12)
C2—C3—C4—C5	−1.1 (7)	C1—O1—Mn1—O4	−2.9 (10)
C3—C4—C5—C6	−0.4 (6)	C1—O1—Mn1—O8	164.2 (3)
C4—C5—C6—C1	1.0 (5)	C1—O1—Mn1—N1	−13.6 (3)
C4—C5—C6—C7	−175.3 (4)	C1—O1—Mn1—O6	−106.8 (3)
O1—C1—C6—C5	180.0 (3)	C1—O1—Mn1—O9	78.0 (3)
C2—C1—C6—C5	−0.2 (5)	C15—O8—Mn1—O4	−15.7 (3)
O1—C1—C6—C7	−3.9 (5)	C15—O8—Mn1—O1	165.8 (3)
C2—C1—C6—C7	175.9 (3)	C15—O8—Mn1—N1	−158.1 (13)
C5—C6—C7—N1	171.7 (3)	C15—O8—Mn1—O6	76.5 (3)
C1—C6—C7—N1	−4.5 (5)	C15—O8—Mn1—O9	−103.6 (3)
N1—C9—C10—O3	51.4 (4)	C7—N1—Mn1—O4	−173.7 (3)
C12—C9—C10—O3	−65.0 (3)	C9—N1—Mn1—O4	5.8 (2)
C11—C9—C10—O3	173.0 (3)	C7—N1—Mn1—O1	5.1 (3)
N1—C9—C11—O2	54.4 (4)	C9—N1—Mn1—O1	−175.4 (2)
C12—C9—C11—O2	167.1 (3)	C7—N1—Mn1—O8	−30.9 (16)
C10—C9—C11—O2	−70.3 (4)	C9—N1—Mn1—O8	148.6 (14)
N1—C9—C12—O4	42.2 (3)	C7—N1—Mn1—O6	94.4 (3)
C10—C9—C12—O4	164.4 (3)	C9—N1—Mn1—O6	−86.1 (2)
C11—C9—C12—O4	−73.1 (3)	C7—N1—Mn1—O9	−85.3 (3)
C6—C7—N1—C9	−177.5 (3)	C9—N1—Mn1—O9	94.2 (2)
C6—C7—N1—Mn1	1.9 (4)	C13—O6—Mn1—O4	26.9 (3)
C12—C9—N1—C7	152.4 (3)	C13—O6—Mn1—O1	−159.5 (3)
C10—C9—N1—C7	32.1 (4)	C13—O6—Mn1—O8	−73.4 (3)
C11—C9—N1—C7	−91.0 (3)	C13—O6—Mn1—N1	109.7 (3)
C12—C9—N1—Mn1	−27.1 (3)	C13—O6—Mn1—O9	−74.0 (11)
C10—C9—N1—Mn1	−147.4 (2)	C12—O4—Mn2—O4^i^	150 (48)
C11—C9—N1—Mn1	89.5 (3)	Mn1—O4—Mn2—O4^i^	−47 (48)
C2—C1—O1—Mn1	−164.9 (2)	C12—O4—Mn2—O7^i^	−23.4 (2)
C6—C1—O1—Mn1	14.9 (4)	Mn1—O4—Mn2—O7^i^	139.47 (13)
C9—C12—O4—Mn1	−41.2 (3)	C12—O4—Mn2—O7	156.6 (2)
C9—C12—O4—Mn2	122.8 (2)	Mn1—O4—Mn2—O7	−40.53 (13)
O6—C13—O5—Mn2	−9.9 (6)	C12—O4—Mn2—O5	−113.9 (2)
C14—C13—O5—Mn2	169.9 (3)	Mn1—O4—Mn2—O5	48.97 (13)
O5—C13—O6—Mn1	3.4 (5)	C12—O4—Mn2—O5^i^	66.1 (2)
C14—C13—O6—Mn1	−176.4 (3)	Mn1—O4—Mn2—O5^i^	−131.03 (13)
O8—C15—O7—Mn2	−15.7 (5)	C15—O7—Mn2—O4^i^	−148.5 (3)
C16—C15—O7—Mn2	163.0 (2)	C15—O7—Mn2—O4	31.5 (3)
O7—C15—O8—Mn1	3.5 (5)	C15—O7—Mn2—O7^i^	112 (100)
C16—C15—O8—Mn1	−175.3 (2)	C15—O7—Mn2—O5	−59.7 (3)
C12—O4—Mn1—O1	9.0 (9)	C15—O7—Mn2—O5^i^	120.3 (3)
Mn2—O4—Mn1—O1	−155.3 (8)	C13—O5—Mn2—O4^i^	166.7 (3)
C12—O4—Mn1—O8	−157.9 (2)	C13—O5—Mn2—O4	−13.3 (3)
Mn2—O4—Mn1—O8	37.79 (13)	C13—O5—Mn2—O7^i^	−104.3 (3)
C12—O4—Mn1—N1	19.8 (2)	C13—O5—Mn2—O7	75.7 (3)
Mn2—O4—Mn1—N1	−144.53 (13)	C13—O5—Mn2—O5^i^	30 (100)
C12—O4—Mn1—O6	112.8 (2)		

Symmetry codes: (i) −*x*+1, −*y*+2, −*z*+2.

### Hydrogen-bond geometry (Å, °)

**Table:**

*D*—H···*A*	*D*—H	H···*A*	*D*···*A*	*D*—H···*A*
O9—H9A···O10^ii^	0.85 (1)	1.97 (1)	2.806 (4)	167 (3)
O9—H9B···O8^iii^	0.85 (1)	2.23 (3)	3.008 (3)	153 (5)
O9—H9B···O1^iii^	0.85 (1)	2.61 (3)	3.322 (3)	142 (5)
O10—H10C···O5^iv^	0.85 (1)	2.06 (1)	2.907 (4)	176 (5)
O10—H10D···N2^v^	0.85 (1)	2.07 (1)	2.914 (6)	174 (6)
O2—H2···O3^vi^	0.82	2.55	3.362 (5)	172.
O3—H3···O6^vii^	0.82	2.00	2.777 (3)	159.

Symmetry codes: (ii) *x*, *y*+1, *z*; (iii) −*x*, −*y*+2, −*z*+2; (iv) −*x*+1, −*y*+1, −*z*+2; (v) *x*, −*y*+3/2, *z*+1/2; (vi) −*x*, *y*−1/2, −*z*+3/2; (vii) −*x*, *y*+1/2, −*z*+3/2.
